# Prepregnancy obesity is associated with cognitive outcomes in boys in a low-income, multiethnic birth cohort

**DOI:** 10.1186/s12887-019-1853-4

**Published:** 2019-12-20

**Authors:** Elizabeth M. Widen, Amy R. Nichols, Linda G. Kahn, Pam Factor-Litvak, Beverly J. Insel, Lori Hoepner, Sara M. Dube, Virginia Rauh, Frederica Perera, Andrew Rundle

**Affiliations:** 1 Department of Nutritional Sciences, College of Natural Sciences, University of Texas at Austin, 103 W 24TH ST A2703, Austin, TX 78712 USA; 2 Columbia Center for Children’s Environmental Health, Mailman School of Public Health, Columbia University, 722 West 168th Street, 12th Floor, New York, NY 10032 USA; 3 Department of Pediatrics, New York University School of Medicine, 403 East 34th St, New York, NY 10016 USA; 4 Department of Epidemiology, Mailman School of Public Health, Columbia University, 722 West 168 Street Room 1614, New York, NY 10032 USA; 5 Department of Environmental Health Sciences, Mailman School of Public Health, Columbia University, New York, NY USA; 6 Department of Environmental and Occupational Health Sciences, SUNY Downstate Medical Center, School of Public Health, 450 Clarkson Avenue, MSC 43, Brooklyn, NY 11203 USA; 7 Department of Nutritional Sciences, 1400 Barbara Jordan Blvd, Austin, TX 78723 USA; 8 Heilbrunn Department of Population and Family Health, Mailman School of Public Health, Columbia University, 60 Haven Avenue, B-2, Room 213, New York, NY 10032 USA

**Keywords:** Body mass index, Cognition, Pregnancy, Child, Overweight, Obesity, Prospective studies, Wechsler scales

## Abstract

**Background:**

Maternal obesity and high gestational weight gain (GWG) disproportionally affect low-income populations and may be associated with child neurodevelopment in a sex-specific manner. We examined sex-specific associations between prepregnancy BMI, GWG, and child neurodevelopment at age 7.

**Methods:**

Data are from a prospective low-income cohort of African American and Dominican women (*n* = 368; 44.8% male offspring) enrolled during the second half of pregnancy from 1998 to 2006. Neurodevelopment was measured using the Wechsler Intelligence Scale for Children (WISC-IV) at approximately child age 7. Linear regression estimated associations between prepregnancy BMI, GWG, and child outcomes, adjusting for race/ethnicity, marital status, gestational age at delivery, maternal education, maternal IQ and child age.

**Results:**

Overweight affected 23.9% of mothers and obesity affected 22.6%. At age 7, full-scale IQ was higher among girls (99.7 ± 11.6) compared to boys (96.9 ± 13.3). Among boys, but not girls, prepregnancy overweight and obesity were associated with lower full-scale IQ scores [overweight β: − 7.1, 95% CI: (− 12.1, − 2.0); obesity β: − 5.7, 95% CI: (− 10.7, − 0.7)]. GWG was not associated with full-scale IQ in either sex.

**Conclusions:**

Prepregnancy overweight and obesity were associated with lower IQ among boys, but not girls, at 7 years. These findings are important considering overweight and obesity prevalence and the long-term implications of early cognitive development.

## Background

Low-income, urban children are at higher risk of not achieving their developmental potential [1–3]. Furthermore, low-income, multiethnic populations are disproportionally affected by adverse prenatal factors, such as excessive maternal adiposity and high gestational weight gain (GWG) [4, 5]. Prior studies suggest that prepregnancy body mass index (BMI) and/or GWG may be negatively associated with cognitive development in early and mid-childhood [6–14]; however, these associations have not been examined in a low-income, multiethnic urban population.

Fetal development depends on maternal nutrition status, but the systemic inflammation, metabolic stress, and hormonal perturbations that accompany excess adiposity may adversely affect placental function and fetal development at critical phases [15–17]. While child sex is a determinant of behavior and cognition, and evidence suggests that boys and girls respond differently to adverse exposures (e.g., poverty, stress, prenatal lead exposure [18, 19]), the interplay among maternal BMI and/or GWG, child sex and cognitive development is poorly understood. We recently reported differences in associations of maternal prepregnancy BMI and child development by sex in our cohort at age 3; specifically we found that maternal obesity was associated with lower psychomotor development index scores in boys, but not girls [20]. Whether these sex-specific effects persist into mid-childhood remains unknown.

Child growth and development are also shaped by environmental and socioeconomic factors, many of which are interrelated. Although partially heritable, child cognition may be predicted by postnatal aspects of the home environment, such as parental nurturance or environmental stimulation [21, 22]. A more nurturing environment has the potential to temper adverse effects from other key determinants, such as limited socioeconomic resources, environmental exposures and possibly maternal excess adiposity or GWG; however, this has not been evaluated [12, 23, 24]. Environmental toxicant exposures, including pesticides and air pollution, are associated with child neural development [25–29] and have been linked to weight and fat mass gain [30–35]. Because pregnancy includes shifts in adipose tissue depots [36], toxicant exposure levels in utero could potentially vary by prepregnancy BMI and GWG; but it is unclear if toxicants impact associations between BMI and/or GWG and child cognition.

Therefore, among low-income African American and Dominican urban children participating in the Columbia Center for Children’s Environmental Health (CCCEH) Mothers and Newborns Study, we examined whether maternal prepregnancy BMI and GWG were related to neurodevelopment at child age 7 and if associations varied by child sex. We hypothesized that maternal obesity and greater GWG would be associated with lower IQ, and that associations would be stronger among boys. Moreover, we evaluated whether a more nurturing postnatal home environment changed directions of associations. We also conducted a sensitivity analysis to evaluate whether associations observed were moderated or confounded by prenatal exposure to chlorpyrifos (CPF) and polycyclic aromatic hydrocarbons (PAH), which were previously associated with decreased child IQ in our population [25, 26].

## Methods

This analysis was conducted in a subset of a cohort designed to examine the role of environmental exposures on birth outcomes. Since 1997, the CCCEH Mothers and Newborns cohort (*n* = 727) has followed mother-child dyads from northern Manhattan and the South Bronx, previously described in detail [37]. From 1997 to 2006, Dominican and African American women with singleton gestations were enrolled from prenatal clinics at New York Presbyterian Medical Center and Harlem Hospital if they met eligibility criteria, including first prenatal visit < 20 weeks of gestation and no self-reported diabetes, hypertension, HIV, illicit drug use or smoking during pregnancy.

An initial prenatal visit during the second or third trimester included maternal measurements and an interviewer-administered questionnaire. Self-reported prepregnancy weight, Income, marital status, exposure to environmental tobacco smoke, and prenatal distress, including demoralization (i.e. psychological stress) [38], use of public assistance, and material hardship (self-report of challenges affording food, paying utilities) [39] were assessed. Self-reported height was obtained at the prenatal visit, and measured height was obtained at postnatal follow-up visits. Maternal height data checking and cleaning in this cohort was previously described in detail [40].

After delivery, medical records were abstracted to ascertain prenatal medical history, last measured weight prior to delivery and infant birth weight. Total GWG was calculated by subtracting the last measured weight prior to delivery from the self-reported prepregnancy weight. BMI category-specific gestational-age standardized weight gain Z-scores (GWG Z-scores) were calculated from total GWG, as previously described, for women with last measured prenatal weights within 4 weeks of delivery [41, 42]. Positive GWG Z-scores indicate that GWG is above average for a gestational age duration, and negative Z-scores indicate that GWG is below average for a given gestational age. For tests of interaction, we used the GWG-Z score calculated using the normal weight women reference for all participants, and for other tests BMI-category specific Z-scores were used. Maternal intelligence was assessed with the Test of Nonverbal Intelligence (2nd edition) (TONI), a 15-min language-free measure of general intelligence, at child age 3 years during a follow up visit at our testing center. During a home visit, at mean child age 3.6 years (range 1.1–6.3 years), a trained researcher conducted the 1-h unstructured Home Observation for Measurement of the Environment (HOME) Inventory to assess learning materials, language stimulation, academic stimulation, variety, and parental responsivity, modeling and acceptance [28]. At child age 7, the Wechsler Intelligence Scale for Children (WISC-IV) was administered by a trained bilingual research assistant. Ten WISC-IV subscales were used for this study [29]. Raw scores were converted into scaled scores, as previously described, and scales scores were derived into composite scores assessing four cognitive indices: verbal comprehension, perceptual reasoning, working memory and processing speed). The composite scores were summed to yield a full-scale composite IQ score. Average expected performance on WISC-IV is a score of 100 (with a standard deviation of 15), and intellectual disability is typically defined as a WISC-IV full-scale IQ score less than or equal to 70.

This study was approved by the Institutional Review Board at Columbia University. Informed consent was obtained from all participating mothers and assent was obtained from the children at age 7.

Analyses were conducted with Stata 14.0 (Stata-Corp, College Station, TX, USA) using an alpha of 0.05 and 0.1 for statistical tests of a priori hypotheses and interactions, respectively.

A complete-case analysis was conducted. Baseline characteristics were compared using chi-square tests, t-tests, and Wilcoxon rank-sum tests. ANOVA was used to compare mean characteristics across prepregnancy BMI categories by child sex. Standard BMI categories were used to allow for comparisons with other reports, and withour findings at age 3 [20]. Multivariable linear regression was used to evaluate associations of 1) maternal prepregnancy BMI category and 2) prepregnancy BMI category and GWG Z-score [41, 42] with child continuous WISC-IV full-scale IQ and index specific scores.

Potential confounders and effect modifiers were identified by causal diagrams and literature review. Potential effect modifiers of the associations between prepregnancy BMI and child outcome, included child sex and GWG. First, we evaluated if associations between prepregnancy BMI category and child IQ varied by child sex on the additive scale by including an interaction term between BMI and sex. We observed effect modification by sex, so all subsequent models were sex-stratified. Then, we included interaction terms between GWG and prepregnancy BMI category to examine effect modification by GWG Z-score on the additive scale. Potential confounders included maternal race/ethnicity (Dominican or African American), marital status (yes/no, married or cohabitating), education (≥high school vs. <high school), age (continuous), parity (nulliparous vs. parous), maternal IQ (continuous), demoralization (total score > 1.55, representing the highest quartile of demoralization in the sample) and hardship (yes/no, defined as at least 1 unmet basic need: going without food, shelter, utilities or clothing at least once during pregnancy). Potential confounders were retained in the model if they changed the beta coefficient for BMI category by > 10%. The final adjustment set included maternal race/ethnicity, marital status, education and maternal IQ, plus child gestational age at delivery (weeks) and age at testing (months) to reduce variance in the outcome. We investigated the postnatal HOME score (continuous) by adding this factor to the primary model and examining change in beta coefficients.

An additional sensitivity analysis examined whether inclusion of the common environmental toxicants chlorpyrifos (CPF) and polycyclic aromatic hydrocarbons (PAH), collected as part of the original study design, modified or confounded associations (See Additional file 1 for details).

Despite strategies designed to improve retention in this longitudinal study [43], a number of participants lacked outcome data due to loss to follow-up by child age 7. To address this, inverse probability weighting (IPW) was used to assess effects of attrition, as previously conducted in this cohort [44]. Separately for boys and girls, a logistic regression model was fit with baseline data, including maternal prepregnancy BMI, parity, age, race/ethnicity, education and hardship, predicting successful retention from which a predicted probability was estimated, and the inverse of this probability was used as a sampling weight in the re-analysis of the linear models.

## Results

**Fig. 1:**
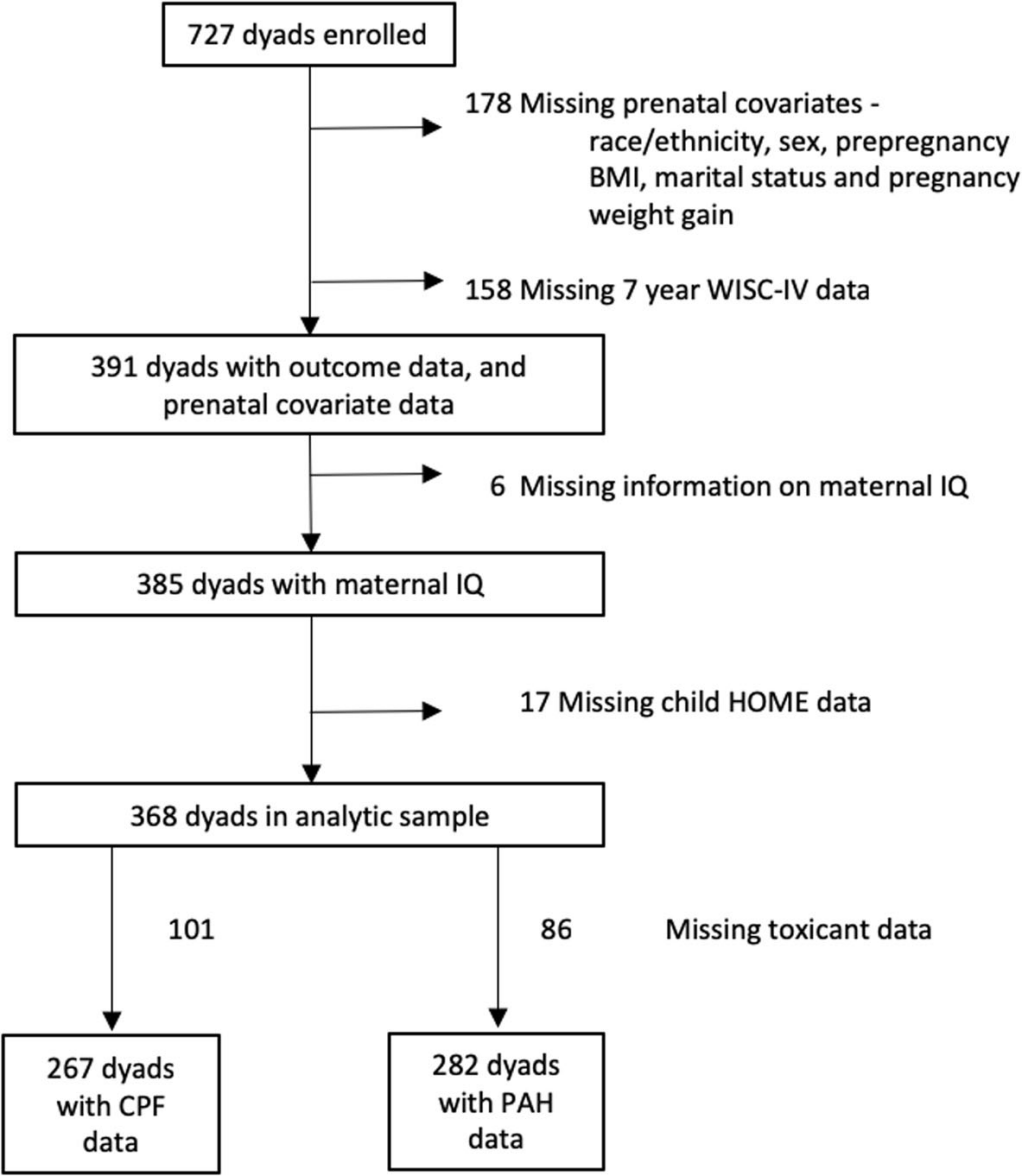
Participant flow diagram

From the original cohort (*n* = 727), complete data were available on 368 dyads (Fig. 1). Baseline characteristics were similar between included and excluded dyads (data not shown); however, compared to those not included, the relative proportion of African American dyads included was higher (41.3 vs. 28.4%) and Dominican dyads was lower (58.7 vs. 71.6%).

**Fig. 2:**
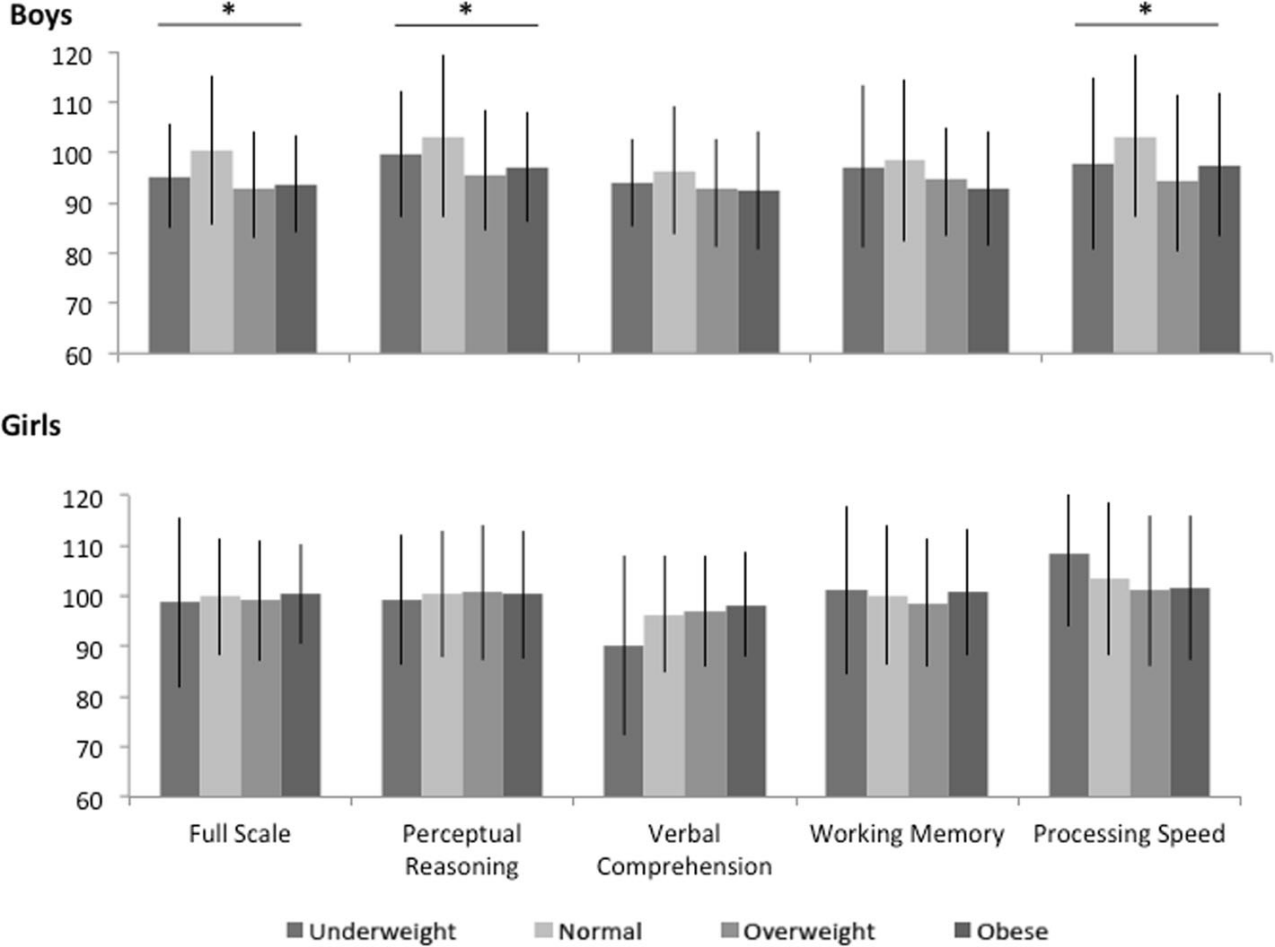
Mean values (Mean ± SD) for child WISC-IV scores at age 7 by sex and prepregnancy BMI category. *Indicate that scores vary across prepregnancy BMI categories

Among all mothers, average total GWG was 16.5 ± 7.4 kg (Mean ± SD) and GWG Z-score was 0.16 ± − 3.6. Table 1 shows baseline characteristics and child measures by sex. At child age 7, full-scale IQ and working memory scores were higher among girls compared to boys. Unadjusted mean values for WISC-IV scores by prepregnancy BMI and child sex are outlined in Fig. 2. In boys, perceptual reasoning, full-scale IQ, and processing speed scores varied by prepregnancy BMI category, with higher scores found among boys born to women with normal prepregnancy BMI values (see Additional fi1e 1: Table S1). Scores did not vary by prepregnancy BMI in girls.

**Table 1:** Participant demographics and outcome values by child sex (*n* = 368)

	Boys (n = 165)	Girls (n = 203)	p-value
Maternal			
Prepregnancy BMI category, n (%)			0.54
Underweight	9 (5.5)	9 (4.4)	
Normal weight	83 (50.3)	95 (46.8)	
Overweight	34 (20.6)	55 (27.1)	
Obese	39 (23.6)	44 (21.7)	
Dominican ethnicity, n (%)	98 (59.4)	118 (58.1)	0.81
Maternal education <high school, n (%)	49 (29.7)	78 (38.4)	0.08
Receipt of public assistance or Medicaid, n (%)^a^	152 (92.7)	183 (90.6)	0.48
Never married, %	108 (65.5)	143 (70.4)	0.31
HOME score	38.8 ± 6.5	39.7 ± 6.0	0.14
Total GWG, kg	17.0 ± 6.8	16.1 ± 7.9	0.25
GWG Z-score	0.26 ± 0.95	0.08 ± 1.09	0.10
Maternal IQ score	85.0 ± 13.1	86.4 ± 13.3	0.35
Detectable PAH^c^, n (%)	48 (36.9)	55 (36.2)	0.89
High chlorpyrifos^d^ (> 6.17 pg/g), n (%)	19 (16.1)	18 (12.1)	0.34
Child			
Age at WISC-IV, months	84.6 ± 2.1	84.8 ± 2.3	0.42
Full-scale composite WISC-IV score	96.9 ± 13.3	99.7 ± 11.6	0.03
Intellectual disability^e^, n (%)	1 (0.61)	3 (1.5)	0.42
Verbal comprehension WISC-IV score	94.6 ± 11.9	96.6 ± 11.5	0.11
Perceptual reasoning WISC-IV score	100.0 ± 14.5	100.3 ± 12.7	0.78
Working memory WISC-IV score	96.3 ± 14.2	99.9 ± 13.4	0.01
Processing speed WISC-IV score	99.8 ± 16.2	102.6 ± 14.9	0.08

Values are means ± SD or percentages. ^a^Data available on 152 boys and 183 girls; ^c^Data available on 130 boys and 152 girls; ^d^Data available on 118 boys and 149 girls; ^e^Full-scale WISC-IV score < =70. BMI, body mass index; GWG, Gestational weight gain; HOME, Home Observation for Measurement of the Environment; PAH, polycyclic aromatic hydrocarbons; WISC-IV, Wechsler Intelligence Scale for Children

In our multivariable models, the association between prepregnancy BMI and child cognitive outcomes varied by sex. Specifically, the interaction *p*-values between prepregnancy overweight or obesity and infant sex were 0.06 and 0.09, respectively, for full-scale IQ. This suggests that associations between prepregnancy BMI and child IQ were different among girls compared to boys. As full-scale IQ is a composite score reflecting four cognitive indices, we sex-stratified subsequent full-scale and index-specific models.

Among boys in our initial multivariable models with and without adjustment for GWG (Table 2 **– Models 1 & 2**), maternal overweight and obesity were associated with lower full-scale IQ and perceptual reasoning scores. Only maternal overweight was associated with lower processing speed scores, and only maternal obesity was associated with lower verbal comprehension scores. Among girls, prepregnancy BMI category was not associated with full-scale IQ or any of the four indices in models with and without adjustment for GWG (Table 2 **– Models 3 & 4**). No interactions between prepregnancy BMI category and GWG were observed within the sex-stratified tables. When GWG z-scores were included as covariates in the sex-stratified models, GWG was not associated with cognitive outcomes in boys. Among girls, however, an inverse association between GWG and perceptual reasoning was observed.

**Table 2:** Adjusted associations between maternal prepregnancy BMI, pregnancy weight gain and child cognitive test scores in boys and girls at age 7, Columbia Center for Children’s Environmental Health, enrolled from 1998 to 2006

	Full Scale		Perceptual Reasoning		Processing Speed		Verbal Comprehension		Working Memory	
	β (95% CI)	p-value	β (95% CI)	p-value	β (95% CI)	p-value	β (95% CI)	p-value	β (95% CI)	p-value
BOYS (n = 165)										
Model 1 Boys: Primary model with BMI										
Prepregnancy BMI										
Underweight	−5.6 (−14.8, 3.7)	0.24	−4.4 (− 14.8, 5.9)	0.40	−8.0 (− 19.6, 3.5)	0.17	0.06 (− 8.2, 8.4)	0.99	− 3.5 (− 13.8, 6.7)	0.50
Normal weight	Reference		Reference		Reference		Reference		Reference	
Overweight	−7.4 (−12.5, −2.3)	0.01	−7.6 (−13.3, − 1.9)	0.01	−8.9 (− 15.3, − 2.5)	0.01	−3.3 (− 7.9, 1.3)	0.15	−3.5 (− 9.1, 2.2)	0.23
Obese	−6.1 (− 11.0, − 1.2)	0.02	−5.5 (− 11.0, − 0.05)	0.05	− 4.6 (− 10.7, 1.5)	0.14	− 4.3 (− 8.7, 0.1)	0.06	−4.8 (− 10.2, 0.6)	0.08
Model 2 Boys: Primary model with BMI, GWG Z-score, without HOME										
Prepregnancy BMI										
Underweight	− 5.9 (− 15.2, 3.4)	0.21	−4.7 (− 15.1, 5.8)	0.38	−8.3 (− 20.0, 3.3)	0.16	−0.3 (− 8.6, 8.1)	0.95	−3.9 (− 14.2, 6.4)	0.46
Normal weight	Reference		Reference		Reference		Reference		Reference	
Overweight	− 7.4 (− 12.5, − 2.3)	0.005	− 7.5 (− 13.3, − 1.8)	0.01	−8.9 (− 15.3, − 2.5)	0.01	−3.3 (− 7.9, 1.3)	0.16	− 3.4 (−9.1, 2.2)	0.23
Obese	−6.5 (− 11.4, − 1.5)	0.01	−5.8 (−11.4, − 0.2)	0.04	−4.9 (− 11.2, 1.3)	0.12	− 4.6 (− 9.1, − 0.2)	0.04	− 5.1 (− 10.7, 0.4)	0.07
GWG Z-score	0.9 (−1.3, 3.0)	0.43	0.6 (−1.8, 3.0)	0.62	0.7 (− 2.0, 3.4)	0.60	0.8 (−1.1, 2.8)	0.39	0.8 (−1.6, 3.2)	0.49
GIRLS (n = 203)										
Model 3 Girls: Primary model with BMI										
Prepregnancy BMI										
Underweight	−1.4 (−9.3, 6,4)	0.72	−1.6 (− 10.4, 7.1)	0.72	3.9 (−6.5, 14.2)	0.46	−6.0 (− 13.3, 1.3)	0.11	1.4 (− 7.9, 10.8)	0.76
Normal weight	Reference		Reference		Reference		Reference		Reference	
Overweight	−1.2 (−5.1, 2.6)	0.53	−0.39 (−4.7, 3.9)	0.86	−2.4 (−7.4, 2.7)	0.35	− 0.20 (−3.8, 3.4)	0.91	−1.2 (− 5.7, 3.4)	0.60
Obese	− 0.74 (− 5.0, 3.5)	0.73	− 0.56 (− 5.3, 4.2)	0.82	−0.62 (−6.3, 5.0)	0.83	− 0.84 (− 4.8, 3.1)	0.68	0.24 (− 4.8, 5.3)	0.93
Model 4 Girls: Primary model with BMI, GWG Z-score without HOME										
Prepregnancy BMI										
Underweight	−1.5 (−9.3, 6.3)	0.70	− 1.7 (− 10.4, 7.0)	0.69	3.8 (−6.5, 14.2)	0.47	−6.0 (− 13.3, 1.3)	0.10	1.4 (− 7.9, 10.6)	0.77
Normal weight	Reference		Reference		Reference		Reference		Reference	
Overweight	−1.5 (−5.3, 2.3)	0.44	−0.74 (− 5.0, 3.5)	0.73	−2.5 (−7.6, 2.6)	0.33	−0.35 (−3.9, 3.2)	0.85	−1.4 (− 5.9, 3.2)	0.55
Obese	−0.6 (−4.9, 3.7)	0.78	−0.4 (− 5.1, 4.4)	0.88	−0.6 (−6.2, 5.1)	0.85	− 0.8 (− 4.7, 3.2)	0.71	0.3 (− 4.7, 5.4)	0.89
GWG Z-score	− 1.3 (− 2.7, 0.2)	0.09	−1.6 (− 3.2, 0.008)	0.05	− 0.5 (− 2.4, 1.4)	0.62	−0.7 (− 2.0, 0.7)	0.31	−0.9 (− 2.6, 0.9)	0.33

Results shown are estimated β-coefficients for WISC-IV composite scores from multivariable linear regression models for each test, controlling for covariates. Normal weight prepregnancy BMI is the reference group. The adjustment set included maternal race/ethnicity, marital status, gestational age at delivery, maternal education, maternal IQ, child age and, for models 2 & 4, the postnatal HOME environment score. IPW, inverse probability weighting. Sample size for each maternal BMI category is as follows for 1) Boys: underweight (n = 9), normal weight (*n* = 83), overweight (*n* = 34), and obese (*n* = 39), and 2) Girls: underweight (*n* = 9), normal weight (*n* = 95), overweight (*n* = 55), and obese (*n* = 44)

In the primary models with additional adjustment for postnatal HOME score (see Additional file 1: Tables S2 & S3 - Model 1), we found that the HOME score impacted several associations, some by > 10%. In boys, BMI category beta coefficients for full-scale IQ and verbal comprehension were attenuated after adjustment for the HOME score. The effect size on full-scale IQ among boys for maternal obesity compared to normal weight was − 6.5 without HOME adjustment, and − 5.7 with HOME adjustment (a 12% difference). Furthermore, the association between maternal obesity and lower verbal comprehension scores in boys no longer existed after adjustment for the HOME score.

Calculation of IPW for retention at child age 7 showed that African American race was associated with follow-up for girls, but not boys, while other factors were not associated with retention (data not shown). Weighting the data did not appreciably alter associations for prepregnancy BMI category models, or for models with both prepregnancy BMI category and GWG (see Additional file 1: Tables S1 & S2 – Models 2 & 3). In toxicant sensitivity analyses, we found no evidence of effect measure modification or confounding of prepregnancy BMI by high CPF or PAH [45] (see Additional file 1).

## Discussion

In this longitudinal cohort of low-income, urban, African American and Dominican maternal-child dyads, we found sex-specific associations in mid-childhood between maternal prepregnancy BMI and child cognitive outcomes in boys, and a limited association between GWG and child perceptual reasoning in girls. Specifically, in boys, maternal overweight and obesity were associated with lower full-scale IQ and perceptual reasoning scores at child age 7, while maternal overweight was associated with lower processing speed scores. Maternal obesity was also associated with lower verbal comprehension scores, although this deficit was attenuated when HOME score was added to the model. The effect sizes for maternal prepregnancy overweight or obesity in boys, when compared to normal weight women, ranged from 4.6 to almost 9 points lower in mid-childhood. Among girls, we observed no association between maternal prepregnancy BMI and cognitive test scores in mid-childhood, but found that gestational-age-standardized GWG was inversely associated with perceptual reasoning after adjustment for the HOME score.

These sex-specific associations observed for effects of maternal excess adiposity on mid-childhood cognitive test scores are intriguing and have not been previously reported. As childhood IQ predicts education level, socioeconomic status and professional success [46], a deficit up to 9 points may be individually meaningful and have implications on a population level. The biological rationale for these observed sex differences in mid-childhood, as well as the biological underpinnings of the links between prepregnancy body size, GWG and child cognitive development, are not fully understood and may be interrelated. Some of the biological pathways linking maternal overweight/obesity and high GWG to fetal and child brain development, structure and function include inflammatory or hormonal perturbations [47–52] and differential dietary or nutrient exposures (e.g., high-fat diet, suboptimal nutrient intakes) [48, 53]. Consistent with previous evidence suggesting that boys are differentially affected by adverse exposures [18], the boys in our study appear to be negatively affected by maternal overweight or obesity compared to girls. Alternatively, the girls could also be adversely affected by maternal overweight or obesity, but in this low-socioeconomic context where boys appear to be more vulnerable to adverse exposures [54] and girls appear to be more responsive or resilient [55], adverse effects on girls’ developmental trajectories may be attenuated by age 7.

The mechanisms underlying these sex-specific findings are unknown, but investigations of explanatory biochemical and molecular changes are ongoing, particularly in the placenta. The placenta mediates fetal programming through regulation of fetal growth and development, and evidence points to sexual dimorphism in placental functioning associated with maternal adiposity [56]. Women with greater adiposity experience greater placental inflammation [57, 58], oxidative and nitrative stress [17, 59] and placental dysfunction compared to women within the normal BMI range [17, 60, 61]. A growing body of animal and human evidence indicates that placental function [62], responsivity [63] and endocrine and neurochemical responses [64], determined by global genome expression and regulation [65–68], the epigenome [62, 69] and response to maternal inflammation and diet [70–72], affect the growing fetus in a sex-specific manner as early as conception [62]. Additionally, males and females develop at different rates in utero [73], and a faster growing fetus has greater exposure to prenatal insults that may partly explain why males are at increased risk for developing adverse pregnancy outcomes [62, 74].

It is challenging to compare our findings to other reports because neurodevelopmental sex differences for maternal pregnancy weight-related exposures have previously not been explored in a low-income, urban population, and further, previous studies examined a wide range of cognitive functions assessed over varying periods of follow-up [75]. However, our findings in boys are consistent with most previous studies in similarly aged children (5-8y) reporting significant associations for prepregnancy BMI alone [6, 11, 13, 76, 77] or prepregnancy BMI and GWG [8, 10, 12, 78]. In girls, we found no associations for prepregnancy BMI and observed an unexpected inverse association for GWG with perceptual reasoning scores when models were adjusted for the HOME score. These findings are less consistent with previous reports where associations for GWG were also observed, but only among women with higher prepregnancy weight or BMI [8, 14, 79].

The quality of the home environment and parenting practices in childhood are important contributors to child neurodevelopment, and the role of a stimulating and nurturing environment on associations may vary by child sex [24, 28]. We do not believe that any previous similar study evaluated whether the postnatal home environment impacted associations. In separate studies, Farah et al. in children ages 4 and 8 and Horton and Kahn et al. in children at 7 years found that parental nurturance predicted child working memory; additionally, Horton and Kahn et al. found that boys benefited more than female counterparts from a nurturing home environment [22]. In building our models, we found that a stimulating and nurturing postnatal home environment attenuated associations between prepregnancy BMI and child cognitive scores in some models. This suggests that the home environment may be on the causal pathway, as posited by Farah and in animal models [22], or a positive confounder between maternal pregnancy weight-related factors and child cognition. Therefore, supporting a healthy home environment during pregnancy and thereafter may be an important area for future investigation and intervention.

In our sensitivity analysis, the toxicants CPF and PAH did not modify or confound associations between prepregnancy BMI and child cognitive test scores. While our exposure assessment in cord blood may capture a significant period of exposure near the end of pregnancy (e.g., PAH DNA adducts have an estimated half-life of 3–4 months), this does not reflect the entire course of pregnancy, or the early pregnancy period where the adverse effects of environmental exposures or high prepregnancy BMI and associated inflammation may be stronger [45].

These findings add to the growing evidence that maternal adiposity affects offspring cognition in middle childhood, but there are limitations to this work. First, our sample size may have been underpowered to detect effect measure modification, especially after sex-stratification. Second, as with most studies in this area, we used self-reported prepregnancy weight to calculate prepregnancy BMI, which potentially biased findings [80]; however, we conducted data cleaning on women with longitudinal prenatal weight data and excluded highly implausible values. We had too few women with severe obesity (BMI > 40 kg/m^2^) to evaluate obesity subgroups. This cohort was predominately enrolled in late pregnancy and included women with relatively healthy pregnancies who did not report diabetes or other medical conditions; however, we were unable to account for preeclampsia, gestational diabetes or other conditions in our analyses since these were not abstracted in the original study design. Although there was attrition, we conducted IPW analyses to assess whether attrition biased our findings and the results were essentially unchanged. The strengths of this study include our ability to account for many factors in our analyses, including maternal IQ, the postnatal home environment and, in a subset, urban environmental toxicant exposures. We also used gestational-age-standardized GWG Z-scores to examine GWG, which allowed for assessment of associations independent of gestational age at delivery.

## Conclusions

In summary, we found that prepregnancy overweight and obesity were associated with lower IQ scores in boys at 7 years of age, but not in girls, an association that was partially attenuated by adjustment for the home environment. These sex-specific associations may reflect differences in the intrauterine environment or potentially the postnatal environment, but the mechanisms are currently not well understood. These findings are important in light of the high prevalence of maternal overweight and obesity, and the longer-term implications of early cognitive development.

## Supplementary information

**Additional file 1: Table S1.** Unadjusted mean values (Mean ± SD) for GWG and WISC-IV by child sex and prepregnancy BMI category. **Table S2.** Adjusted associations between maternal prepregnancy BMI, pregnancy weight gain and child cognitive test scores in boys (*n* = 165) at age 7, Columbia Center for Children’s Environmental Health, enrolled from 1998 to 2006. **Table S3.** Adjusted associations between maternal prepregnancy BMI, pregnancy weight gain and child cognitive test scores in girls (*n* = 203) at age 7, Columbia Center for Children’s Environmental Health, enrolled from 1998 to 2006.

## Data Availability

The data for this the current study was used under a limited use data use agreement between Columbia University and the University of Texas at Austin. The data that support the findings of this study are available from Columbia University; but restrictions apply to the availability of these data because of the need to maintain participant confidentiality. Data are available upon request and review by the Columbia Center for Children’s Environmental Health through an institutional data use agreement.

## Abbreviations

BMI: Body mass index
CCCEH: Columbia Center for Children’s Environmental Health
CPF: Chlorpyrifos
GWG: Gestational weight gain
HOME: Home Observation for Measurement of the Environment
IPW: Inverse probability weighting
IQ: Intelligence quotient
PAH: Polycyclic aromatic hydrocarbon
TONI: Test of Nonverbal Intelligence
