# Supplementary material for: Prepregnancy obesity is associated with cognitive outcomes in boys in a low-income, multiethnic birth cohort
Source: BMC Pediatr. 2019 Dec 20;19:507. doi: 10.1186/s12887-019-1853-4 (PMC6924019; doi:10.1186/s12887-019-1853-4)
Supplement: Supplementary file 1 — Additional file 1: Table S1. Unadjusted mean values (Mean ± SD) for GWG and WISC-IV by child sex and prepregnancy BMI category. Table S2. Adjusted associations between maternal prepregnancy BMI, pregnancy weight gain and child cognitive test scores in boys (n = 165) at age 7, Columbia Center for Children’s Environmental Health, enrolled from 1998 to 2006. Table S3. Adjusted associations between maternal prepregnancy BMI, pregnancy weight gain and child cognitive test scores in girls (n = 203) at age 7, Columbia Center for Children’s Environmental Health, enrolled from 1998 to 2006. [file 12887_2019_1853_MOESM1_ESM.pdf]

Additional File 1

Table S1. Unadjusted mean values (Mean±SD) for GWG and WISC-IV by child sex and prepregnancy BMI category.

	**Prepregnancy BMI Category**				
	Underweight	Normal	Overweight	Obese	p-value
**Boys (n=165)**	n=9	n=83	n=34	n=39	
Total GWG, kg	19.4±7.5	17.3±6.5	15.9±4.8	16.7±8.7	0.52
GWG Z-score	0.64±1.0	0.15±1.1	0.08±0.63	0.54±0.78	0.06
WISC-IV score					
Full Scale	95.3±10.3	100.4±14.9	92.7±11.6	93.7±9.7	0.01
Perceptual Reasoning	99.6±12.5	103.2±16.1	95.4±13.0	97.2±11.0	0.03
Verbal Comprehension	94.0±8.7	96.4±12.8	93.0±9.9	92.5±11.8	0.27
Working Memory	97.2±16.1	98.5±16.2	94.8±10.1	92.8±11.4	0.19
Processing Speed	97.8±17.1	103.3±16.1	94.4±16.9	97.5±14.2	0.04
**Girls (n=203)**	n=9	n=95	n=55	n=44	
Total GWG, kg	15.4±4.5	17.6±7.5	15.4±7.6	13.8±9.1	0.06
GWG Z-score	0.02±0.82	0.14±1.16	-0.11±1.10	0.20±1.01	0.50
WISC-IV score					
Full Scale	98.7±17.0	99.9±11.7	99.1±12.1	100.3±9.9	0.95
Perceptual Reasoning	99.1±12.9	100.4±12.6	100.6±13.3	100.3±12.7	0.99
Verbal Comprehension	90.1±17.9	96.3±11.6	96.9±11.1	98.3±10.3	0.27
Working Memory	101.1±16.7	100.1±13.9	98.6±12.8	100.8±12.7	0.86
Processing Speed	108.4±14.4	103.4±15.3	101.0±14.9	101.7±14.4	0.49

Table S2. Adjusted associations between maternal prepregnancy BMI, pregnancy weight gain and child cognitive test scores in boys (n=165) at age 7, Columbia Center for Children's Environmental Health, enrolled from 1998 to 2006.

	Full Scale		Perceptual Reasoning		Processing Speed		Verbal Comprehension		Working Memory	
	β (95% CI)	p-value	β (95% CI)	p-value	β (95% CI)	p-value	β (95% CI)	p-value	β (95% CI)	p-value
**Model 1: Primary model with HOME**										
Prepregnancy BMI										
Underweight	-4.7 (-14.0, 4.6)	0.32	-4.5 (-15.1, 6.0)	0.4	-8.3 (-20.1, 3.5)	0.17	1.4 (-6.8, 9.7)	0.73	-2.4 (-12.6, 7.9)	0.65
Normal weight	Reference		Reference		Reference		Reference		Reference	
Overweight	-7.1 (-12.1, -2.0)	0.01	-7.5 (-13.3, -1.8)	0.01	-8.9 (-15.3, -2.5)	0.01	-2.9 (-7.3, 1.6)	0.21	-3.1 (-8.7, 2.5)	0.28
Obese	-5.7 (-10.7, -0.7)	0.03	-5.7 (-11.4, -0.02)	0.05	-4.9 (-11.3, 1.4)	0.13	-3.6 (-8.1, 0.8)	0.11	-4.3 (-9.8, 1.3)	0.13
GWG Z-score	0.6 (-1.6, 2.7)	0.59	0.6 (-1.9, 3.0)	0.65	0.7 (-2.0, 3.4)	0.60	0.5 (-1.4, 2.4)	0.63	0.5 (-1.9, 2.9)	0.68
**Model 2 IPW: Prepregnancy BMI category**										
Underweight	-3.5 (-10.5, 3.5)	0.32	-4.1 (-11.7, 3.5)	0.29	-5.2 (-19.3, 8.9)	0.47	2.1 (-2.9, 7.1)	0.41	-1.8 (-11.4, 7.8)	0.72
Normal weight	Reference		Reference		Reference		Reference		Reference	
Overweight	-6.8 (-11.6, -2.0)	0.01	-7.1 (-12.7, -1.5)	0.01	-8.7 (-15.2, -2.3)	0.01	-2.9 (-6.8, 1.0)	0.15	-2.8 (-7.7, 2.1)	0.27
Obese	-4.8 (-9.0, -0.5)	0.02	-4.8 (-9.6, 0.06)	0.05	-4.3 (-9.7, 1.1)	0.12	-3.1 (-7.5, 1.3)	0.016	-3.3 (-8.3, 1.8)	0.21
**Model 3 IPW: BMI category and GWG Z-score**										
Prepregnancy BMI										
Underweight	-3.9 (-10.7, 3.0)	0.27	-4.4 (-12.0, 3.2)	0.25	-5.6 (-19.4, 8.2)	0.43	1.8 (-2.9, 6.4)	0.46	-2.1 (-11.7, 7.6)	0.67
Normal weight	Reference		Reference		Reference		Reference		Reference	
Overweight	-6.8 (-11.5, -2.0)	0.01	-7.0 (-12.6, -1.4)	0.01	-8.7 (-15.2, -2.2)	0.01	-2.8 (-6.7, 1.1)	0.15	-2.8 (-7.7, 2.1)	0.27
Obese	-5.1 (-9.4, -0.8)	0.02	-5.1 (-9.9, -0.2)	0.04	-4.7 (-10.4, 1.1)	0.11	-3.4 (-7.7, 1.0)	0.13	-3.5 (-8.7, 1.7)	0.18
GWG Z-score	0.7 (-1.4, 2.8)	0.52	0.6 (-1.5, 2.8)	0.58	0.8 (-2.1, 3.6)	0.60	0.7 (-1.2, 2.5)	0.49	0.6 (-2.1, 3.3)	0.67

Results shown are estimated β-coefficients for WISC-IV composite scores from multivariable linear regression models for each test, controlling for covariates. Normal weight prepregnancy BMI is the reference group. The adjustment set included maternal race/ethnicity, marital status, gestational age at delivery, maternal education, maternal IQ, child age and the postnatal HOME score. IPW, inverse probability weighting. Sample size for each BMI category is as follows: underweight (n=9), normal weight (n=83), overweight (n=34) and obese (n=39).

Table S3. Adjusted associations between maternal prepregnancy BMI, pregnancy weight gain and child cognitive test scores in girls (n=203) at age 7, Columbia Center for Children's Environmental Health, enrolled from 1998 to 2006.

	Full Scale		Perceptual Reasoning		Processing Speed		Verbal Comprehension		Working Memory	
	β (95% CI)	p-value	β (95% CI)	p-value	β (95% CI)	p-value	β (95% CI)	p-value	β (95% CI)	p-value
**Model 1: Primary model with BMI, GWG & HOME**										
Prepregnancy BMI										
Underweight	-0.8 (-8.7, 7.0)	0.83	-1.2 (-10.0, 7.6)	0.79	3.1 (-7.3, 13.6)	0.56	-4.7 (-11.9, 2.6)	0.21	1.7 (-7.7, 11.1)	0.72
Normal weight	Reference		Reference		Reference		Reference		Reference	
Overweight	-1.3 (-5.1, 2.5)	0.51	-0.6 (-4.8, 3.7)	0.79	-2.7 (-7.8, 2.4)	0.29	0.1 (-3.4, 3.6)	0.97	-1.3 (-5.9, 3.3)	0.58
Obese	-0.2 (-4.5, 4.1)	0.92	-0.1 (-4.9, 4.7)	0.98	-1.0 (-6.7, 4.8)	0.74	0.01 (-3.9, 4.0)	1.00	0.5 (-4.6, 5.7)	0.84
GWG Z-score	-1.3 (-2.8, 0.1)	0.07	-1.7 (-3.3, -0.1)	0.04	-0.4 (-2.3, 1.5)	0.68	-0.9 (-2.2, 0.5)	0.21	-0.9(-2.6, 0.8)	0.31
**Model 2 IPW: Prepregnancy BMI category**										
Underweight	-1.9 (-9.9, 6.1)	0.64	-1.7 (-9.5, 6.1)	0.67	2.2 (-8.0, 12.3)	0.68	-5.3 (-13.4, 2.7)	0.19	0.4 (-8.4, 9.2)	0.93
Normal weight	Reference		Reference		Reference		Reference		Reference	
Overweight	-1.5 (-5.3, 2.4)	0.50	-0.3 (-4.6, 4.0)	0.89	-3.5 (-8.6, 1.6)	0.17	-0.02 (-3.5, 3.4)	0.99	-1.5 (-5.8, 2.9)	0.51
Obese	-0.6 (-4.7, 3.4)	0.76	-0.5 (-5.3, 4.4)	0.85	-0.5 (-5.5, 4.5)	0.84	-0.6 (-4.6, 3.4)	0.76	0.1 (-4.9, 5.1)	0.96
**Model 3 IPW: BMI category and GWG Z-score**										
Prepregnancy BMI										
Underweight	-2.0 (-9.9, 6.0)	0.63	-1.7 (-9.5, 6.1)	0.66	2.1 (-8.0, 12.3)	0.68	-5.4 (-13.4, 2.6)	0.19	0.37 (-8.4, 9.2)	0.93
Normal weight	Reference		Reference		Reference		Reference		Reference	
Overweight	-1.8 (-5.6, 2.1)	0.37	-0.7 (-4.9, 3.5)	0.75	-3.7 (-8.8, 1.5)	0.16	-0.2 (-3.7, 3.3)	0.91	-1.6 (-6.0, 2.7)	0.46
Obese	-0.5 (-4.5, 3.5)	0.81	-0.3 (-5.1, 4.5)	0.91	-0.44 (-5.4, 4.5)	0.86	-0.6 (-4.5, 3.4)	0.79	0.20 (-4.8, 5.2)	0.94
GWG Z-score	-1.3 (-2.8, 0.2)	0.10	-1.6 (-3.3, -0.02)	0.05	-0.6 (-2.5, 1.3)	0.56	-0.8 (-2.2, 0.59)	0.26	-0.8 (-2.5, 0.9)	0.37

Results shown are estimated β-coefficients for WISC-IV composite scores from multivariable linear regression models for each test, controlling for covariates. Normal weight prepregnancy BMI is the reference group. The adjustment set included maternal race/ethnicity, marital status, gestational age at delivery, maternal education, maternal IQ, child age and the postnatal HOME score. IPW, inverse probability weighting. Sample size for each BMI category is as follows: underweight (n=9), normal weight (n=95), overweight (n=55) and obese (n=44).

Environmental toxicant exposure sensitivity analyses:

Because we had previously shown that prenatal exposure to the organophosphate insecticide chlorpyrifos (CPF) and polycyclic aromatic hydrocarbons (PAH) were associated with decreased child IQ [1, 2], and in animal models these chemicals are linked to weight gain and fat mass gain in mice [3], we conducted a sensitivity analysis to evaluate whether associations observed were moderated or confounded by prenatal PAH or CPF exposure. We theorized that exposure to these toxicants could affect prepregnancy BMI and also weight changes across pregnancy, which include shifts in overall and regional adipose tissue depots. Levels of fetal exposure to these lipophilic toxicants may vary as adipose depots mobilize during pregnancy.

CPF and PAH were measured in umbilical cord plasma via assays that have been previously described.[1, 2] In the subset of participants with PAH and CPF data, we fit separate models with adjustment for high PAH (detectable/not detectable) or high CPF (>6.17 pg/g, representing the highest quartile). These cutpoints selected were previously used in the papers evaluating associations between these factors and child IQ in our cohort.[1, 2] We explored potential effect measure modification by prepregnancy BMI with each of the toxicants; interaction terms with continuous prepregnancy BMI were used, as we had some small cell sizes (<5) with categorical BMI exposure. If effect measure modification was not observed, we evaluated potential confounding by examining whether the prepregnancy BMI category beta coefficient changed by >10% with adjustment for each of the toxicants in comparison to models without adjustment in the same subsample.

In the toxicant sensitivity analyses, we found no evidence of effect measure modification of prepregnancy BMI by high CPF or high PAH (all BMI-toxicant p-values >0.1). Further, inclusion of high CPF or high PAH in our models did not change the beta coefficients for GWG or prepregnancy BMI category by more than 10% (data not shown), so neither exposure showed evidence of confounding these associations.

References:

1. Perera FP, Rauh V, Whyatt RM, Tsai WY, Tang D, Diaz D, et al. Effect of prenatal exposure to airborne polycyclic aromatic hydrocarbons on neurodevelopment in the first 3 years of life among inner-city children. *Environ Health Perspect*. 2006; 114:1287-1292.

2. Rauh V, Arunajadai S, Horton M, Perera F, Hoepner L, Barr DB, et al. Seven-year neurodevelopmental scores and prenatal exposure to chlorpyrifos, a common agricultural pesticide. *Environ Health Perspect*. 2011; 119:1196-1201.

3. Meggs WJ, Brewer KL. Weight gain associated with chronic exposure to chlorpyrifos in rats. *J Med Toxicol*. 2007; 3:89-93.
